# Direct evidence for sequence-dependent attraction between double-stranded DNA controlled by methylation

**DOI:** 10.1038/ncomms11045

**Published:** 2016-03-22

**Authors:** Jejoong Yoo, Hajin Kim, Aleksei Aksimentiev, Taekjip Ha

**Affiliations:** 1 Department of Physics and the Center for the Physics of Living Cells, University of Illinois at Urbana-Champaign, Urbana, Illinois 61801, USA; 2 School of Life Sciences, Ulsan National Institute of Science and Technology, Ulsan, Korea; 3 Center for Soft and Living Matter, Institute for Basic Science, Ulsan, Korea; 4 Beckman Institute for Advanced Science and Technology, University of Illinois at Urbana-Champaign, Urbana, Illinois 61801, USA; 5 Howard Hughes Medical Institute, Baltimore, Maryland 21205, USA; 6 Department of Biophysics and Biophysical Chemistry, Johns Hopkins University, Baltimore, Maryland 21205, USA; 7 Department of Biophysics, Johns Hopkins University, Baltimore, Maryland 21205, USA; 8 Department of Biomedical Engineering, Johns Hopkins University, Baltimore, Maryland 21205, USA

## Abstract

Although proteins mediate highly ordered DNA organization *in vivo*, theoretical studies suggest that homologous DNA duplexes can preferentially associate with one another even in the absence of proteins. Here we combine molecular dynamics simulations with single-molecule fluorescence resonance energy transfer experiments to examine the interactions between duplex DNA in the presence of spermine, a biological polycation. We find that AT-rich DNA duplexes associate more strongly than GC-rich duplexes, regardless of the sequence homology. Methyl groups of thymine acts as a steric block, relocating spermine from major grooves to interhelical regions, thereby increasing DNA–DNA attraction. Indeed, methylation of cytosines makes attraction between GC-rich DNA as strong as that between AT-rich DNA. Recent genome-wide chromosome organization studies showed that remote contact frequencies are higher for AT-rich and methylated DNA, suggesting that direct DNA–DNA interactions that we report here may play a role in the chromosome organization and gene regulation.

## Abstract (web summary)

Theoretical studies suggest that homologous DNA duplexes can preferentially associate with one another in the absence of proteins. Here, the authors show that GC-rich DNA with methylated cytosine and AT-rich DNA duplexes associate more strongly than GC-rich duplexes regardless of the sequence homology.

## Graphical abstract

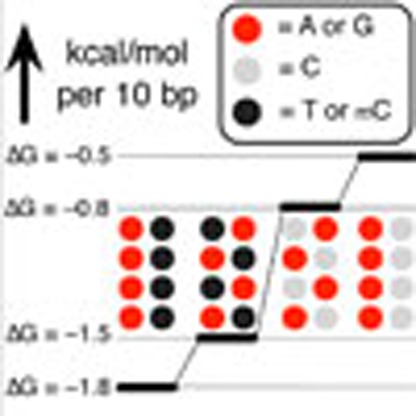

Formation of a DNA double helix occurs through Watson–Crick pairing mediated by the complementary hydrogen bond patterns of the two DNA strands and base stacking. Interactions between double-stranded (ds)DNA molecules in typical experimental conditions containing mono- and divalent cations are repulsive^1^, but can turn attractive in the presence of high-valence cations^2^. Theoretical studies have identified the ion–ion correlation effect as a possible microscopic mechanism of the DNA condensation phenomena^3^,^4^,^5^. Theoretical investigations have also suggested that sequence-specific attractive forces might exist between two homologous fragments of dsDNA^6^, and this ‘homology recognition' hypothesis was supported by *in vitro* atomic force microscopy^7^ and *in vivo* point mutation assays^8^. However, the systems used in these measurements were too complex to rule out other possible causes such as Watson–Crick strand exchange between partially melted DNA or protein-mediated association of DNA.

Here we present direct evidence for sequence-dependent attractive interactions between dsDNA molecules that neither involve intermolecular strand exchange nor are mediated by proteins. Further, we find that the sequence-dependent attraction is controlled not by homology—contradictory to the ‘homology recognition' hypothesis^6^—but by a methylation pattern. Unlike the previous *in vitro* study that used monovalent (Na^+^) or divalent (Mg^2+^) cations^7^, we presumed that for the sequence-dependent attractive interactions to operate polyamines would have to be present. Polyamine is a biological polycation present at a millimolar concentration in most eukaryotic cells and essential for cell growth and proliferation^9^,^10^. Polyamines are also known to condense DNA in a concentration-dependent manner^2^,^11^. In this study, we use spermine^4+^ (Sm^4+^) that contains four positively charged amine groups per molecule.

## Results

### Sequence dependence of DNA–DNA forces

**Figure 1:**
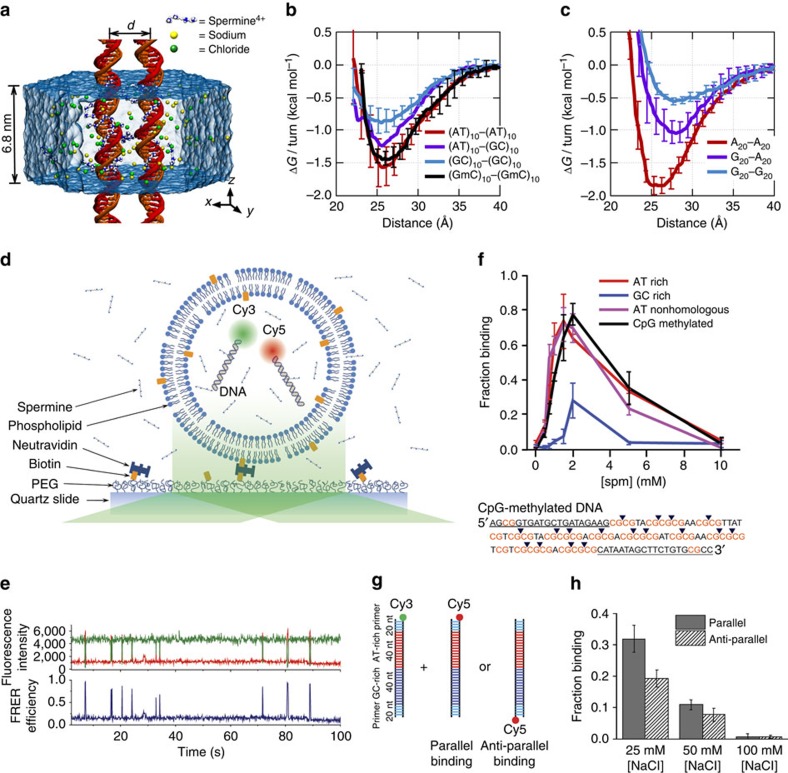
Polyamine-mediated DNA sequence recognition observed in MD simulations and smFRET experiments. (**a**) Set-up of MD simulations. A pair of parallel 20-bp dsDNA duplexes is surrounded by aqueous solution (semi-transparent surface) containing 20 Sm^4+^ molecules (which compensates exactly the charge of DNA) and 100 mM NaCl. Under periodic boundary conditions, the DNA molecules are effectively infinite. A harmonic potential (not shown) is applied to maintain the prescribed distance between the dsDNA molecules. (**b**,**c**) Interaction free energy of the two DNA helices as a function of the DNA–DNA distance for repeat-sequence DNA fragments (**b**) and DNA homopolymers (**c**). (**d**) Schematic of experimental design. A pair of 120-bp dsDNA labelled with a Cy3/Cy5 FRET pair was encapsulated in a ∼200-nm diameter lipid vesicle; the vesicles were immobilized on a quartz slide through biotin–neutravidin binding. Sm^4+^ molecules added after immobilization penetrated into the porous vesicles. The fluorescence signals were measured using a total internal reflection microscope. (**e**) Typical fluorescence signals indicative of DNA–DNA binding. Brief jumps in the FRET signal indicate binding events. (**f**) The fraction of traces exhibiting binding events at different Sm^4+^ concentrations for AT-rich, GC-rich, AT nonhomologous and CpG-methylated DNA pairs. The sequence of the CpG-methylated DNA specifies the methylation sites (CG sequence, orange), restriction sites (BstUI, triangle) and primer region (underlined). The degree of attractive interaction for the AT nonhomologous and CpG-methylated DNA pairs was similar to that of the AT-rich pair. All measurements were done at [NaCl]=50 mM and *T*=25 °C. (**g**) Design of the hybrid DNA constructs: 40-bp AT-rich and 40-bp GC-rich regions were flanked by 20-bp common primers. The two labelling configurations permit distinguishing parallel from anti-parallel orientation of the DNA. (**h**) The fraction of traces exhibiting binding events as a function of NaCl concentration at fixed concentration of Sm^4+^ (1 mM). The fraction is significantly higher for parallel orientation of the DNA fragments.

To characterize the molecular mechanisms of DNA–DNA attraction mediated by polyamines, we performed molecular dynamics (MD) simulations where two effectively infinite parallel dsDNA molecules, 20 base pairs (bp) each in a periodic unit cell, were restrained to maintain a prescribed inter-DNA distance; the DNA molecules were free to rotate about their axes. The two DNA molecules were submerged in 100 mM aqueous solution of NaCl that also contained 20 Sm^4+^ molecules; thus, the total charge of Sm^4+^, 80 *e*, was equal in magnitude to the total charge of DNA (2 × 2 × 20 *e*, two unit charges per base pair; Fig. 1a). Repeating such simulations at various inter-DNA distances and applying weighted histogram analysis^12^ yielded the change in the interaction free energy (Δ*G*) as a function of the DNA–DNA distance (Fig. 1b,c). In a broad agreement with previous experimental findings^13^, Δ*G* had a minimum, Δ*G*_min_, at the inter-DNA distance of 25−30 Å for all sequences examined, indeed showing that two duplex DNA molecules can attract each other. The free energy of inter-duplex attraction was at least an order of magnitude smaller than the Watson–Crick interaction free energy of the same length DNA duplex. A minimum of Δ*G* was not observed in the absence of polyamines, for example, when divalent or monovalent ions were used instead^14^,^15^.

Unexpectedly, we found that DNA sequence has a profound impact on the strength of attractive interaction. The absolute value of Δ*G* at minimum relative to the value at maximum separation, |Δ*G*_min_|, showed a clearly rank-ordered dependence on the DNA sequence: |Δ*G*_min_| of (A)_20_>|Δ*G*_min_| of (AT)_10_>|Δ*G*_min_| of (GC)_10_>|Δ*G*_min_| of (G)_20_. Two trends can be noted. First, AT-rich sequences attract each other more strongly than GC-rich sequences^16^. For example, |Δ*G*_min_| of (AT)_10_ (1.5 kcal mol^−1^ per turn) is about twice |Δ*G*_min_| of (GC)_10_ (0.8 kcal mol^−1^ per turn) (Fig. 1b). Second, duplexes having identical AT content but different partitioning of the nucleotides between the strands (that is, (A)_20_ versus (AT)_10_ or (G)_20_ versus (GC)_10_) exhibit statistically significant differences (∼0.3 kcal mol^−1^ per turn) in the value of |Δ*G*_min_|.

To validate the findings of MD simulations, we performed single-molecule fluorescence resonance energy transfer (smFRET)^17^ experiments of vesicle-encapsulated DNA molecules. Equimolar mixture of donor- and acceptor-labelled 120-bp dsDNA molecules was encapsulated in sub-micron size, porous lipid vesicles^18^ so that we could observe and quantitate rare binding events between a pair of dsDNA molecules without triggering large-scale DNA condensation^2^. Our DNA constructs were long enough to ensure dsDNA–dsDNA binding that is stable on the timescale of an smFRET measurement, but shorter than the DNA's persistence length (∼150 bp (ref. 19)) to avoid intramolecular condensation^20^. The vesicles were immobilized on a polymer-passivated surface, and fluorescence signals from individual vesicles containing one donor and one acceptor were selectively analysed (Fig. 1d). Binding of two dsDNA molecules brings their fluorescent labels in close proximity, increasing the FRET efficiency (Fig. 1e).

FRET signals from individual vesicles were diverse. Sporadic binding events were observed in some vesicles, while others exhibited stable binding; traces indicative of frequent conformational transitions were also observed (Supplementary Fig. 1A). Such diverse behaviours could be expected from non-specific interactions of two large biomolecules having structural degrees of freedom. No binding events were observed in the absence of Sm^4+^ (Supplementary Fig. 1B) or when no DNA molecules were present. To quantitatively assess the propensity of forming a bound state, we chose to use the fraction of single-molecule traces that showed any binding events within the observation time of 2 min (Methods). This binding fraction for the pair of AT-rich dsDNAs (AT1, 100% AT in the middle 80-bp section of the 120-bp construct) reached a maximum at ∼2 mM Sm^4+^ (Fig. 1f), which is consistent with the results of previous experimental studies^2^,^3^. In accordance with the prediction of our MD simulations, GC-rich dsDNAs (GC1, 75% GC in the middle 80 bp) showed much lower binding fraction at all Sm^4+^ concentrations (Fig. 1b,c). Regardless of the DNA sequence, the binding fraction reduced back to zero at high Sm^4+^ concentrations, likely due to the resolubilization of now positively charged DNA–Sm^4+^ complexes^2^,^3^,^13^.

Because the donor and acceptor fluorophores were attached to the same sequence of DNA, it remained possible that the sequence homology between the donor-labelled DNA and the acceptor-labelled DNA was necessary for their interaction^6^. To test this possibility, we designed another AT-rich DNA construct AT2 by scrambling the central 80-bp section of AT1 to remove the sequence homology (Supplementary Table 1). The fraction of binding traces for this nonhomologous pair of donor-labelled AT1 and acceptor-labelled AT2 was comparable to that for the homologous AT-rich pair (donor-labelled AT1 and acceptor-labelled AT1) at all Sm^4+^ concentrations tested (Fig. 1f). Furthermore, this data set rules out the possibility that the higher binding fraction observed experimentally for the AT-rich constructs was caused by inter-duplex Watson–Crick base pairing of the partially melted constructs.

Next, we designed a DNA construct named ATGC, containing, in its middle section, a 40-bp AT-rich segment followed by a 40-bp GC-rich segment (Fig. 1g). By attaching the acceptor to the end of either the AT-rich or GC-rich segments, we could compare the likelihood of observing the parallel binding mode that brings the two AT-rich segments together and the anti-parallel binding mode. Measurements at 1 mM Sm^4+^ and 25 or 50 mM NaCl indicated a preference for the parallel binding mode by ∼30% (Fig. 1h). Therefore, AT content can modulate DNA–DNA interactions even in a complex sequence context. Note that increasing the concentration of NaCl while keeping the concentration of Sm^4+^ constant enhances competition between Na^+^ and Sm^4+^ counterions, which reduces the concentration of Sm^4+^ near DNA and hence the frequency of dsDNA–dsDNA binding events (Supplementary Fig. 2).

### Methylation determines the strength of DNA–DNA attraction

**Figure 2:**
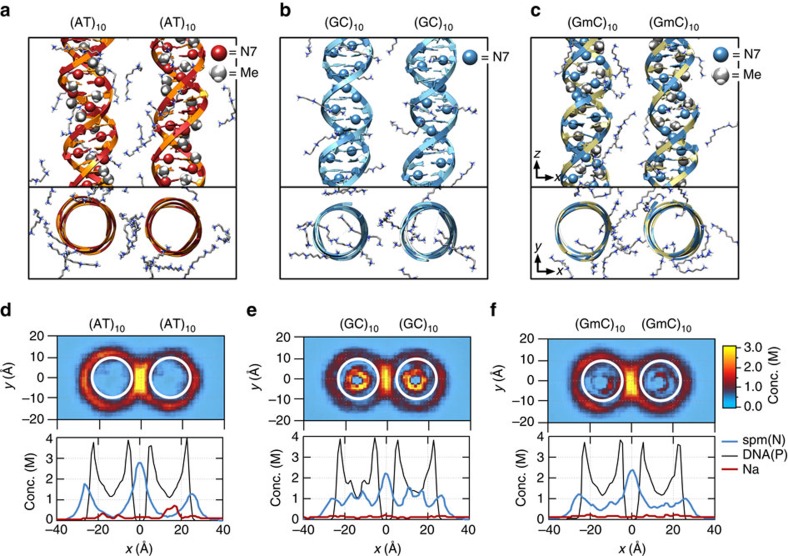
Molecular mechanism of polyamine-mediated DNA sequence recognition. (**a**–**c**) Representative configurations of Sm^4+^ molecules at the DNA–DNA distance of 28 Å for the (AT)_10_–(AT)_10_ (**a**), (GC)_10_–(GC)_10_ (**b**) and (GmC)_10_–(GmC)_10_ (**c**) DNA pairs. The backbone and bases of DNA are shown as ribbon and molecular bond, respectively; Sm^4+^ molecules are shown as molecular bonds. Spheres indicate the location of the N7 atoms and the methyl groups. (**d**–**f**) The average distributions of cations for the three sequence pairs featured in **a**–**c**. Top: density of Sm^4+^ nitrogen atoms (*d*=28 Å) averaged over the corresponding MD trajectory and the *z* axis. White circles (20 Å in diameter) indicate the location of the DNA helices. Bottom: the average density of Sm^4+^ nitrogen (blue), DNA phosphate (black) and sodium (red) atoms projected onto the DNA–DNA distance axis (*x* axis). The plot was obtained by averaging the corresponding heat map data over *y*=[−10, 10] Å. See Supplementary Figs 4 and 5 for the cation distributions at *d*=30, 32, 34 and 36 Å.

Analysis of the MD simulations revealed the molecular mechanism of the polyamine-mediated sequence-dependent attraction (Fig. 2). In the case of the AT-rich fragments, the bulky methyl group of thymine base blocks Sm^4+^ binding to the N7 nitrogen atom of adenine, which is the cation-binding hotspot^21^,^22^. As a result, Sm^4+^ is not found in the major grooves of the AT-rich duplexes and resides mostly near the DNA backbone (Fig. 2a,d). Such relocated Sm^4+^ molecules bridge the two DNA duplexes better, accounting for the stronger attraction^16^,^23^,^24^,^25^. In contrast, significant amount of Sm^4+^ is adsorbed to the major groove of the GC-rich helices that lacks cation-blocking methyl group (Fig. 2b,e).

If indeed the extra methyl group in thymine, which is not found in cytosine, is responsible for stronger DNA–DNA interactions, we can predict that cytosine methylation, which occurs naturally in many eukaryotic organisms and is an essential epigenetic regulation mechanism^26^, would also increase the strength of DNA–DNA attraction. MD simulations showed that the GC-rich helices containing methylated cytosines (mC) lose the adsorbed Sm^4+^ (Fig. 2c,f) and that |Δ*G*_min_| of (GC)_10_ increases on methylation of cytosines to become similar to |Δ*G*_min_| of (AT)_10_ (Fig. 1b).

To experimentally assess the effect of cytosine methylation, we designed another GC-rich construct GC2 that had the same GC content as GC1 but a higher density of CpG sites (Supplementary Table 1). The CpG sites were then fully methylated using M. SssI methyltransferase (Supplementary Fig. 3; Methods). As predicted from the MD simulations, methylation of the GC-rich constructs increased the binding fraction to the level of the AT-rich constructs (Fig. 1f).

**Figure 3:**
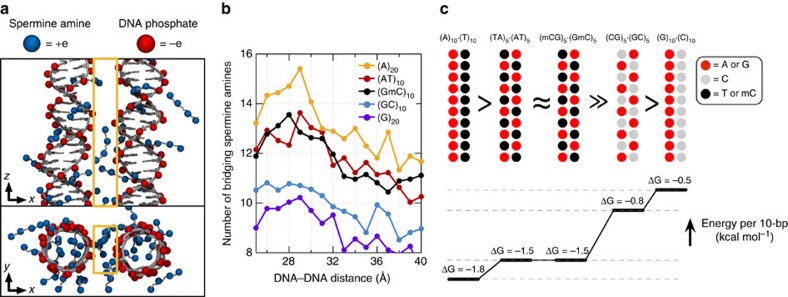
Methylation modulates the interaction free energy of two dsDNA molecules by altering the number of bridging Sm^4+^. (**a**) Typical spatial arrangement of Sm^4+^ molecules around a pair of DNA helices. The phosphates groups of DNA and the amine groups of Sm^4+^ are shown as red and blue spheres, respectively. ‘Bridging' Sm^4+^ molecules reside between the DNA helices. Orange rectangles illustrate the volume used for counting the number of bridging Sm^4+^ molecules. (**b**) The number of bridging amine groups as a function of the inter-DNA distance. The total number of Sm^4+^ nitrogen atoms was computed by averaging over the corresponding MD trajectory and the 10 Å (*x* axis) by 20 Å (*y* axis) rectangle prism volume (**a**) centred between the DNA molecules. (**c**) Schematic representation of the dependence of the interaction free energy of two DNA molecules on their nucleotide sequence. The number and spatial arrangement of nucleotides carrying a methyl group (T or mC) determine the interaction free energy of two dsDNA molecules.

The sequence dependence of |Δ*G*_min_| and its relation to the Sm^4+^ adsorption patterns can be rationalized by examining the number of Sm^4+^ molecules shared by the dsDNA molecules (Fig. 3a). An Sm^4+^ cation adsorbed to the major groove of one dsDNA is separated from the other dsDNA by at least 10 Å, contributing much less to the effective DNA–DNA attractive force than a cation positioned between the helices, that is, the ‘bridging' Sm^4+^ (ref. 23). An adsorbed Sm^4+^ also repels other Sm^4+^ molecules due to like-charge repulsion, lowering the concentration of bridging Sm^4+^. To demonstrate that the concentration of bridging Sm^4+^ controls the strength of DNA–DNA attraction, we computed the number of bridging Sm^4+^ molecules, *N*_spm_ (Fig. 3b). Indeed, the number of bridging Sm^4+^ molecules ranks in the same order as |Δ*G*_min_|: *N*_spm_ of (A)_20_>*N*_spm_ of (AT)_10_≈*N*_spm_ of (GmC)_10_>*N*_spm_ of (GC)_10_>*N*_spm_ of (G)_20_. Thus, the number density of nucleotides carrying a methyl group (T and mC) is the primary determinant of the strength of attractive interaction between two dsDNA molecules. At the same time, the spatial arrangement of the methyl group carrying nucleotides can affect the interaction strength as well (Fig. 3c). The number of methyl groups and their distribution in the (AT)_10_ and (GmC)_10_ duplex DNA are identical, and so are their interaction free energies, |Δ*G*_min_| of (AT)_10_≈|Δ*G*_min_| of (GmC)_10_. For AT-rich DNA sequences, clustering of the methyl groups repels Sm^4+^ from the major groove more efficiently than when the same number of methyl groups is distributed along the DNA (Fig. 3b). Hence, |Δ*G*_min_| of (A)_20_>|Δ*G*_min_| of (AT)_10_. For GC-rich DNA sequences, clustering of the cation-binding sites (N7 nitrogen) attracts more Sm^4+^ than when such sites are distributed along the DNA (Fig. 3b), hence |Δ*G*_min_| is larger for (GC)_10_ than for (G)_20_.

## Discussion

Genome-wide investigations of chromosome conformations using the Hi–C technique revealed that AT-rich loci form tight clusters in human nucleus^27^,^28^. Gene or chromosome inactivation is often accompanied by increased methylation of DNA^29^ and compaction of facultative heterochromatin regions^30^. The consistency between those phenomena and our findings suggest the possibility that the polyamine-mediated sequence-dependent DNA–DNA interaction might play a role in chromosome folding and epigenetic regulation of gene expression.

## Methods

### MD protocols

All MD simulations were carried out in a constant-temperature/constant-pressure ensemble using the Gromacs 4.5.5 package^31^. Integration time step was 2 fs. The temperature was set to 300 K using the Nosé–Hoover scheme^32^,^33^. The pressure in the *xy* plane (normal to DNA) was kept constant at 1 bar using the Parrinello–Rahman scheme^34^; the length of the box in the *z* direction was kept constant at 68 Å. Van der Waals forces were evaluated using a 7–10-Å switching scheme. Long-range electrostatic forces were computed using the particle-Mesh Ewald summation scheme^35^, a 1.5-Å Fourier-space grid and a 12-Å cutoff for the real-space Coulomb interaction. Covalent bonds to hydrogen in water, and in non-water molecules were constrained using SETTLE^36^ and LINCS^37^ algorithms, respectively. All simulations were carried out using the AMBER99bsc0 force field for DNA^38^,^39^, NaCl parameters of Joung *et al*.^40^ and the TIP3P water model^41^. Parameters describing spermine (NH_2_(CH_2_)_3_NH(CH_2_)_4_NH(CH_2_)_3_NH_2_) were based on the AMBER99 force field^24^. Custom Van der Waals parameters (CUFIX) were used to describe non-bonded interactions between spermine amine and DNA phosphate, between sodium ion and DNA phosphate, and between sodium and chloride ions^14^,^15^,^25^,^42^.

### Potential of mean force calculations

Initially, a pair of 20-bp duplexes DNA was placed in a hexagonal water box parallel to the *z* axis. The water box measured ∼130 Å within the *xy* plane and 68 Å along the *z* axis. Each DNA strand was effectively infinite under the periodic boundary conditions. The relatively large lateral size of the simulation box was chosen to avoid finite size artefacts. Twenty Sm^4+^ molecules were randomly placed in the box to neutralize the charge of the DNA molecules; sodium and chloride ions were added corresponding to a 100 mM concentration. Five variants of the system were built, different only by the nucleotide sequence of the DNA molecules. Each system was equilibrated for at least 50 ns; the DNA molecules were free to move about the simulation system during the equilibration. The last frame of each equilibration trajectory was used to initiate umbrella sampling simulations that determined the free energy (Δ*G*) of the pair of parallel dsDNA molecules as a function of the inter-DNA distance. The reaction coordinate was defined as the distance between the centres of mass of the two DNA molecules projected onto the *xy* plane. The harmonic restraints used for umbrella sampling simulations had a force constant of 2,000 kJ mol^−1^ nm^−2^; the inter-DNA distance varied from 23 to 42 Å with a 1-Å window spacing. The umbrella sampling simulations were ∼200 ns in duration in each sampling window; the inter-DNA distance was recorded every 2 ps. Except for the umbrella restraining potential, no additional constraints were applied to DNA. The weighted histogram analysis method implemented in the Gromacs package was used for the reconstruction of the free energy from the recorded inter-DNA distance data^12^.

### DNA design and synthesis

Supplementary Table 1 specifies the design of the 120-bp long DNA template and other molecules used in this study. All DNA constructs had the same 20-bp primer regions at both the ends of the constructs (primer A and B, designed by Clone Manager Suite 7). Primer B was labelled at the 5′ end with either Cy3 or Cy5 dye with the efficiency of 90% or higher. The nucleotide sequence of the middle 80-bp section of the constructs varied among the constructs. Note that we did not use 100% GC constructs because they are known to contain quadruplexes^43^. The 120-bp DNA templates were made by Integrated DNA Technologies. The dsDNA constructs were synthesized from these templates and two primers by PCR using Phusion High-Fidelity PCR Master Mix kit (New England BioLabs) and following the standard protocol of the kit. The PCR products were purified using the QIAquick PCR purification kit (Qiagen) and their concentrations were measured by ultraviolet–visible absorption. The CpG-methylated constructs were obtained by performing an 8-h methylation reaction on the dsDNA constructs using the CpG methyltransferase M. SssI (New England BioLabs, M0226L) following the company's standard protocol. The product was purified by the PCR purification kit. The methylation efficiency was estimated by digesting the dsDNA products with the BstUI restriction enzyme (New England BioLabs, R0518L), which can cut only unmethylated CGCG sequence, and subsequent electrophoresis of the digested fragments through a polyacrylamide gel (Supplementary Fig. 3).

### DNA encapsulation

We encapsulated the purified dsDNA in lipid vesicles by modifying the protocol previously developed for single-stranded DNA^18^. In short, we mixed biotinyl cap phosphoethanolamine with 1,2-dimyristoyl-sn-glycero-3-phosphocholine in 1:100 molar ratio, dried and hydrated with buffer solution of 100 mM NaCl, and 25 mM Tris (pH 8.0). After hydration, the mixture was flash-frozen in liquid nitrogen and thawed seven times to create large unilamellar vesicles. The desired pair of dsDNAs was added to the solution, each at 400 nM concentration (corresponding to 1 molecule in a spherical volume of 200 nm diameter) and then the solution was extruded through a membrane filter with 200 nm pores to create uniformly sized unilamellar vesicles. Typical acceptor co-encapsulation yield (defined as the fraction of vesicles detected with a pair of donor and acceptor among all vesicles with any acceptor signal) was 10–20%.

### Single-molecule measurements

The lipid vesicles were immobilized on a PEG-coated surface through the biotin–neutravidin interaction (Fig. 2a). Fluorescence signals from individual vesicles were collected by total internal reflection microscopy as previously described^44^. Imaging solution contained 1 mg ml^−1^ glucose oxidase, 0.04 mg ml^−1^ catalase, 0.8% dextrose, saturated Trolox (∼3 mM) and 25 mM Tris in addition to the desired amounts of NaCl and Sm^4+^. The gel–liquid transition temperature of 1,2-dimyristoyl-sn-glycero-3-phosphocholine is 24 °C, which results in a bistable membrane structure at room temperature, allowing the exchange of ions and small molecules (Sm^4+^, Trolox and so on) through the membrane. All measurements and solution exchanges were carried out at 25 °C. Fluorescence movies were taken with the rate of 100 ms per frame. For Cy3 and Cy5 dyes, 532- and 647-nm solid-state lasers were used as the excitation sources, respectively.

### Analysis of single-molecule traces

We quantified the strength of binding by measuring the fraction of traces that showed any binding events among all traces containing a single pair of donor and acceptor dyes. Binding traces exhibited a variety of behaviours (Supplementary Fig. 1A), which in part can be attributed to the variation in the vesicle size. First, we selected the traces containing single pair of Cy3 and Cy5 by examining their bleaching steps and signal intensities from either excitation. Among these, we selected the traces showing clear binding behaviours. The criteria we used as the binding behaviours was either the FRET efficiency jumping over 0.5 or showing no clear jumps but maintaining a FRET level of 0.25 or higher. The number of these binding traces over the number of all single pair traces was measured for triplicate data sets at each Sm^4+^ concentrations for each DNA sample. The error bars in Fig. 1f represents the s.e. of mean between these triplicate measurements.

## Additional information

**How to cite this article:** Yoo, J. *et al*. Direct evidence for sequence-dependent attraction between double-stranded DNA controlled by methylation. *Nat. Commun.* 7:11045 doi: 10.1038/ncomms11045 (2016).

## Supplementary Material

Supplementary Information Supplementary Figures 1-5 and Supplementary Table 1
