# Supplementary material for: Direct evidence for sequence-dependent attraction between double-stranded DNA controlled by methylation
Source: Nat Commun. 2016 Mar 22;7:11045. doi: 10.1038/ncomms11045 (PMC4804163; doi:10.1038/ncomms11045)
Supplement: Supplementary Information — Supplementary Figures 1-5 and Supplementary Table 1 [file ncomms11045-s1.pdf]

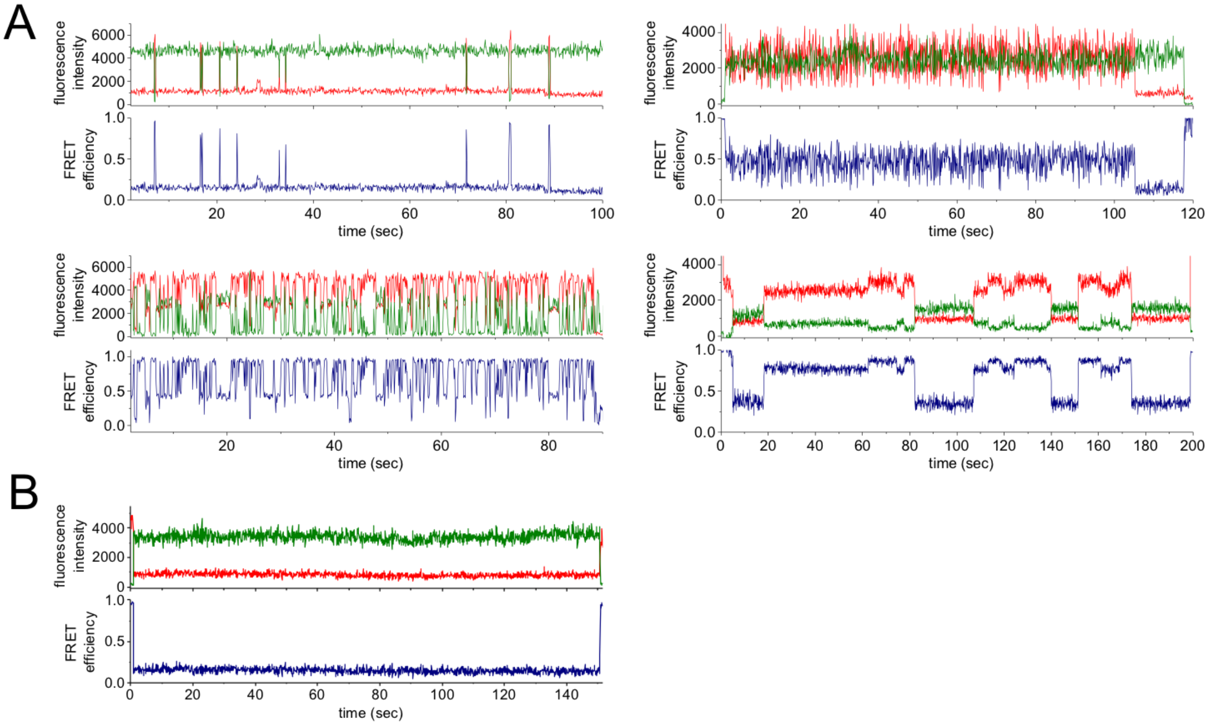

Supplementary Figure 1. Representative FRET signals from vesicle-encapsulated pairs of dsDNA molecules in the presence (A) and absence (B) of spermine. (A) Four representative FRET signals taken at 1 mM spermine and 50 mM NaCl (Cy5 excitation is not displayed). These FRET signals report on various behaviors of the encapsulated pairs of DNA molecules including binding, dissociation, diffusion, rotation, bending, or kinking. The vesicle encapsulation technique makes observations of such early stages of molecular aggregation possible at the single molecular level. To quantify the binding propensity taking into account the heterogeneous behavior of individual FRET pairs, we calculated the fraction of vesicle-encapsulated pairs that exhibited any type of dynamics featuring high FRET efficiency values. The protocol for quantification of binding events is described in detail in Materials and Methods. (B) A representative FRET signal taken in the absence of spermine (50 mM NaCl only) shows no binding events. Cy5 was excited during the first and last 1 second of the measurement to verify the presence of co-encapsulated Cy5-DNA strand.

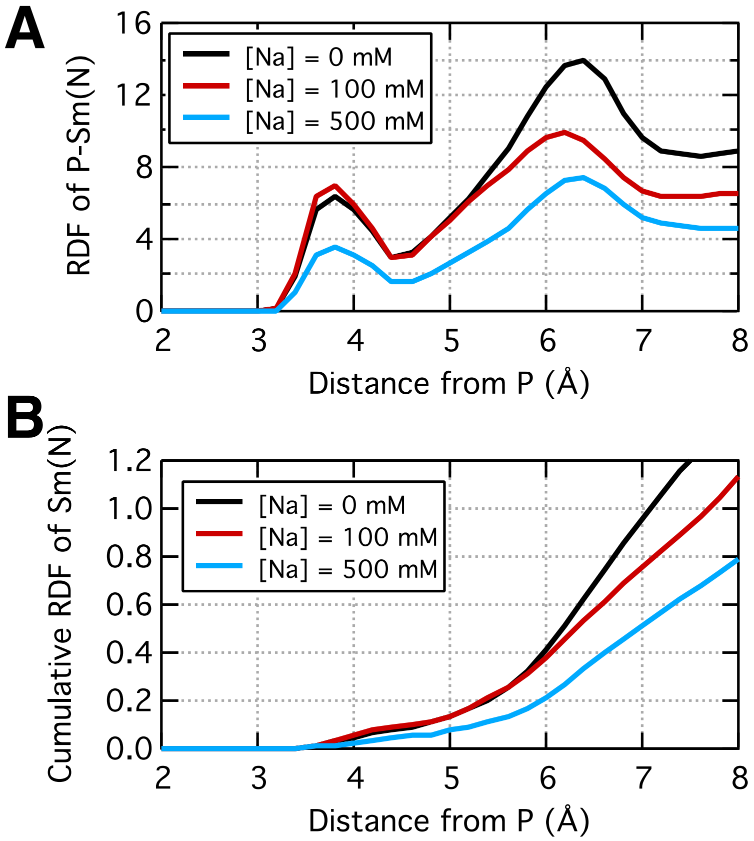

Supplementary Figure 2. The simulated effect of background NaCl concentration on Sm^{4+} binding to DNA. (A,B) Radial distribution function (A) and cumulative radial distribution function (B) of the spermine nitrogen atoms, Sm(N), with respect to the DNA phosphorus atom, P. Data at [Na] = 100 mM were computed using the umbrella sampling trajectory of the AT10 constructs at the inter-DNA distance of 4 nm. Data at [Na] = 0 and 500 mM were obtained by repeating the above umbrella sampling simulation after adjusting the ion concentration in the system; each of the two simulations lasted about 60 ns. All three simulation systems contained the same number (20) of Sm^{4+} molecules and had approximately the same volume.

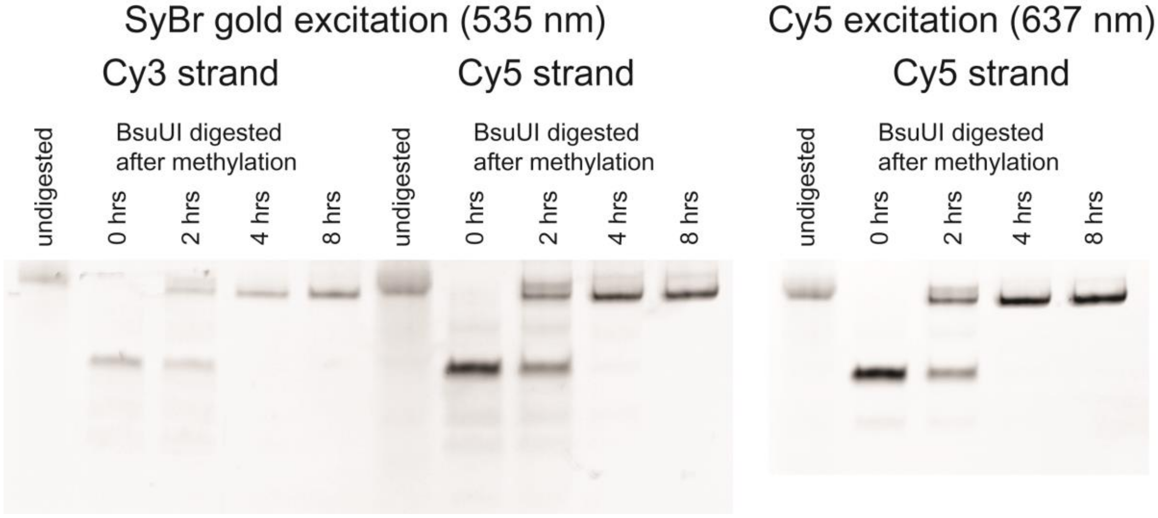

Supplementary Figure 3. Restriction digestion assay of CpG methylated constructs. Our CG2 DNA construct contained 32 CpG sites, including 17 restriction sites (CGCG) of the BstUI digestion enzyme. Supplementary Table S1 lists the complete nucleotide sequence of the construct. A BstUI enzyme cannot digest DNA at the restriction sites that contain methylated cytosine nucleotides. To characterize the efficiency of our cytosine methylation protocol, we varied the duration of the DNA methylation reaction and characterized the reaction products by means of BstUI digestion followed by gel electrophoresis. After methylation but before BstUI digestion DNA molecules were purified using a PCR purification kit. The BstUI digestion was carried out following the protocols from New England BioLabs (Materials and Methods). The figure shows images of the labeled digestion products after electrophoresis through a 15% polyacrylamide gel (left-hand-side: DNA stained, right-hand-side: Cy5 label). The amount of short DNA fragments (produced by BstUI digestion) diminishes as the duration of the DNA methylation reaction increases. No restriction digestion products were detected after four hours of DNA methylation reaction, which indicates complete methylation of all CpG sites of the construct.

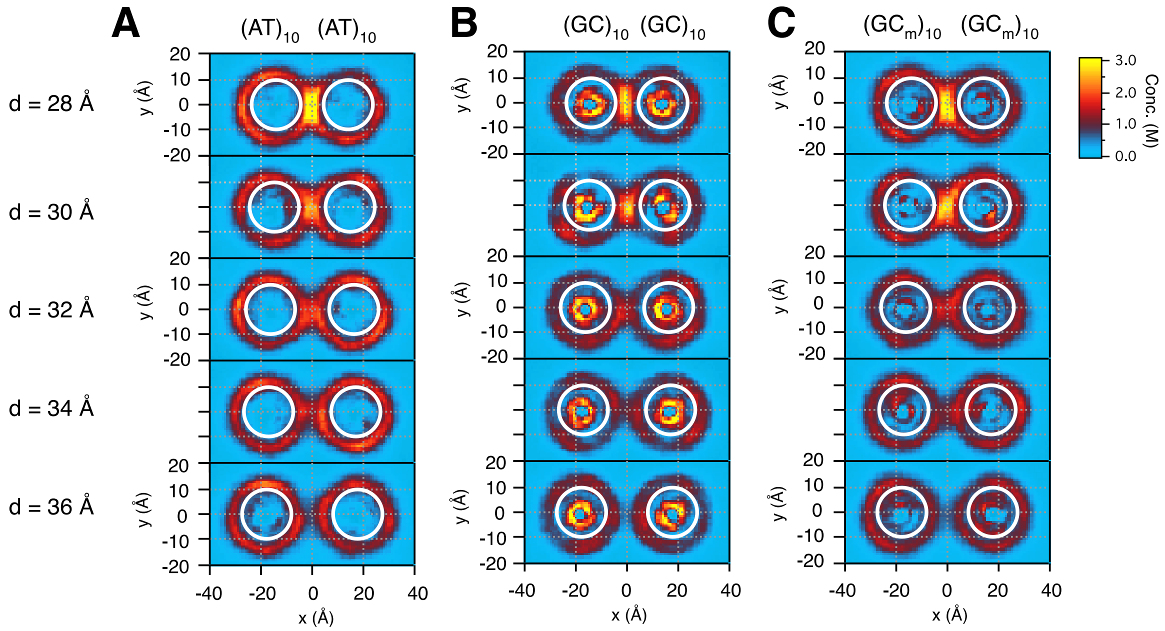

Supplementary Figure 4. Local concentration of spermine around three pairs of dsDNA molecules. Data are shown for the $(AT)_{10}$-$(AT)_{10}$ (panel A); $(GC)_{10}$-$(GC)_{10}$ (panel B); $(GmC)_{10}$-$(GmC)_{10}$ (panel C) sequence pairs at five inter-DNA distances (vertical rows). Each heat map shows the concentration of spermine nitrogen atoms averaged over the *z*-axis (the directions of the DNA molecules) and the corresponding simulation trajectory. White circles (20 Å in diameter) indicate the locations of the DNA helices.

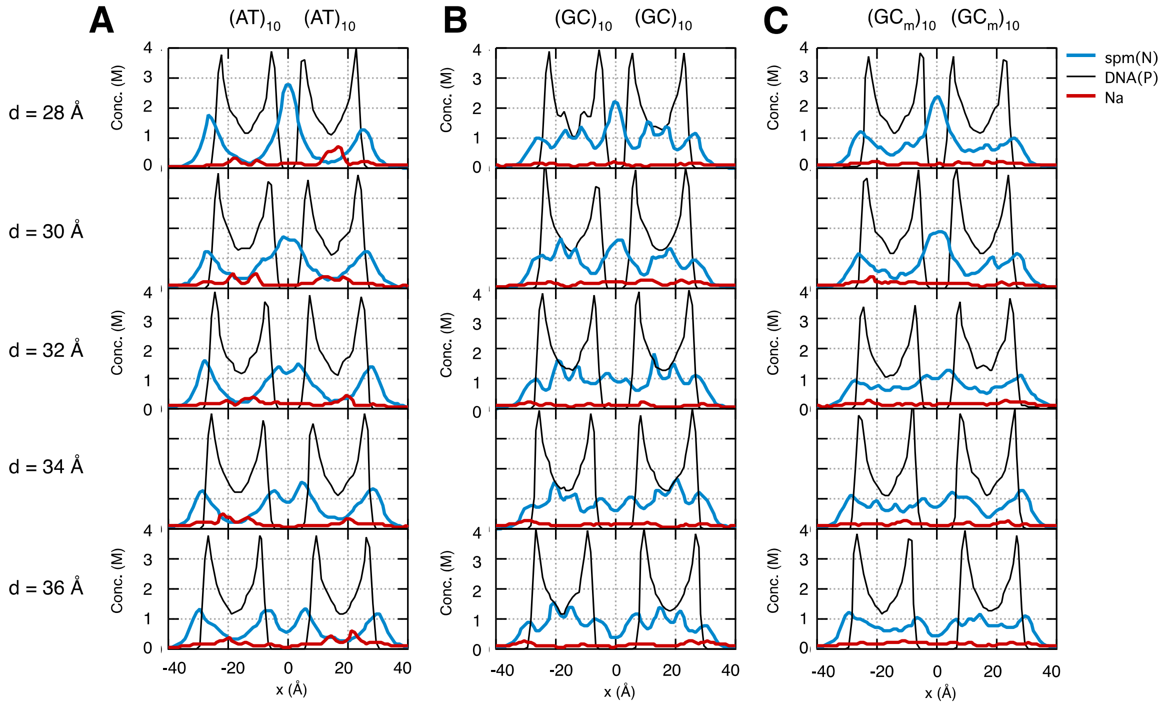

Supplementary Figure 5. The concentration of DNA phosphorous atoms (black), spermine nitrogen atoms (blue) and sodium ions (red) along the DNA-DNA distance axis (*x*-axis). Data are shown for the $(AT)_{10}$-$(AT)_{10}$ (panel A); $(GC)_{10}$-$(GC)_{10}$ (panel B); $(GmC)_{10}$-$(GmC)_{10}$ (panel C) sequence pairs at five inter-DNA distances (vertical rows). The profiles of spermine concentration were obtained by averaging the corresponding heat map data (Supplementary Figure S4) over y = [−10, 10] Å. The same procedures were used to obtain the concentration profiles of sodium ions and DNA phosphorous atoms.

Supplementary Table 1. DNA sequences used in smFRET experiments. An underline denotes a primer region; italic font indicates a CpG methylation site; a dot denotes a BstUI restriction site. The sequence of the non-homologous AT-rich DNA molecule (AT2) does not match the sequence of the AT-rich DNA (AT1) for any DNA fragment of ten nucleotides or longer. The GC-rich DNA molecule used for the CpG methylation experiments (GC2) has the same GC content as the GC-rich DNA molecule (CG1).

Name	Sequence
primer A	5'-AGCGGTGATGCTGATAGAAG-3'
primer B	5'-C6 amine GGCGCACAGAAGCTATTATG-3'
AT-rich DNA (AT1)	5'-AGCGGTGATGCTGATAGAAGTATAATATTAATAATAAATTAAATATATTATATTAATAATTAATAAT<br>TAATAAATTAAAATATTATTTATAATAATTAAACATAATAGCTTCTGTGCGCC-3'
Non-homologous AT-rich DNA (AT2)	5'-AGCGGTGATGCTGATAGAAGATATATAAAATTTAAATTATATAAAATTATTTAATATAATTATTTTT<br>TAAATATAATTTTTAAATAATTTAATATTTAAACATAATAGCT TCTGTGCGCC-3'
GC-rich DNA (GC1)	5'-AGCGGTGATGCTGATAGAAGCGCGACTGCCCGCCGAGATATCCTGGGGCGCAGCGCGGACGGATGT<br>CCCACGGGGTTGCCGCGCGCGCCGAGCGCTTCGCCATAATAGCTTCTGTGCGCC-3'
GC-rich DNA for CpG methylation (GC2)	5'-AG*CG*GTGATGCTGATAGAAG*CG*•*CG*TA*CG*•*CG*•*CG*AA*CG*•*CG*TTAT*CG*T*CG*•*CG*TA*CG*•*CG*•*CG*A*CG*•*C*<br>*G*A*CG*•*CG*•*CG*AT*CG*•*CG*AA*CG*•*CG*•*CG*T*CG*T*CG*•*CG*•*CG*A*CG*•*CG*•*CG*CATAATAGCTTCTGTG*CG*CC-3'
Hybrid GC-AT DNA (ATGC)	5'-AGCGGTGATGCTGATAGAAGCGCGACTGCCCGCCGAGATATCCTGGGGCGCAGCGCGGACTAATAA<br>TTAATAAATTAAAATATTATTTATAATAATTAAACATAATAGCTTCTGTGCGCC-3'
